# Retention in Opioid Agonist Therapy Among First Nations People

**DOI:** 10.1001/jamanetworkopen.2025.18452

**Published:** 2025-06-30

**Authors:** Alice Holton, Bisola Hamzat, Daniel McCormack, Sacha Bragg, Bernadette deGonzague, Graham Mecredy, Tonya Campbell, Tony Antoniou, Lorrilee McGregor, Jonathan Bertram, Tara Gomes

**Affiliations:** 1 Li Ka Shing Knowledge Institute of St. Michael's Hospital, Unity Health Toronto, Toronto, Ontario, Canada; 2 MAP Centre for Urban Health Solutions, St Michael’s Hospital, Toronto, Ontario, Canada; 3 Royal College of Surgeons in Ireland School of Pharmacy and Biomolecular Sciences, Dublin, Ireland; 4 ICES, Toronto, Ontario, Canada; 5 Chiefs of Ontario, Toronto, Ontario, Canada; 6 Department of Family and Community Medicine, Unity Health Toronto, Toronto, Ontario, Canada; 7 Department of Family and Community Medicine, University of Toronto, Toronto, Ontario, Canada; 8 Northern Ontario School of Medicine University, Sudbury, Ontario, Canada; 9 Centre for Addiction and Mental Health, Toronto, Ontario; 10 Leslie Dan Faculty of Pharmacy, University of Toronto, Toronto, Ontario, Canada; 11 Institute for Health Policy, Management and Evaluation, University of Toronto, Toronto, Ontario, Canada

## Abstract

**Importance:**

First Nations people are disproportionately impacted by the opioid crisis in Canada. While many First Nation communities have expanded access to treatment, there is a need to better understand the factors associated with early discontinuation of opioid agonist therapies (OAT).

**Objective:**

To investigate factors associated with OAT retention within the first year of treatment among First Nations people in Ontario, Canada.

**Design, Setting, and Participants:**

This was a population-based retrospective cohort study including all registered (status) First Nations people aged 15 years or older initiating OAT between January 2013 and March 2021. Data were analyzed between October 2022 and June 2024.

**Exposure:**

Methadone and buprenorphine-naloxone initiation.

**Main Outcomes and Measures:**

The main outcome was duration of OAT treatment, with discontinuation defined as a gap in therapy of more than 14 days. Cox proportional hazards models followed up individuals until the first occurrence of OAT discontinuation, death, end of 1-year follow-up, or switching between OAT treatments.

**Results:**

A total of 17 880 OAT initiations among 7476 individuals (median [IQR] age, 31 [26-38] years; 8966 [50.1%] female) were identified, including 9074 new episodes of buprenorphine-naloxone and 8806 new episodes of methadone. Time to treatment discontinuation was shorter among buprenorphine-naloxone episodes (median [IQR], 42 [5-321] days) compared with methadone episodes (median [IQR], 71 [10-544] days) (*P* < .001). Several factors were associated with buprenorphine-naloxone and methadone retention, including living in moderately sized urban areas (buprenorphine-naloxone: adjusted hazard ratio [aHR], 0.81; 95% CI, 0.70-0.95; methadone: aHR, 0.79; 95% CI, 0.70-0.90) and being recently dispensed non-OAT opioids (buprenorphine-naloxone: aHR, 0.86; 95% CI, 0.80-0.94; methadone: aHR, 0.86; 95% CI, 0.79-0.93). In contrast, factors associated with higher rates of discontinuation included recent opioid toxic events (buprenorphine-naloxone: aHR, 1.36; 95% CI, 1.20-1.54; methadone: aHR, 1.24; 95% CI, 1.11-1.38), and recent methadone treatment (buprenorphine-naloxone: aHR, 1.09; 95% CI, 1.01-1.18; methadone: aHR, 1.67; 95% CI, 1.57-1.78). Methadone discontinuation increased over time; however this pattern was not observed for buprenorphine-naloxone.

**Conclusions and Relevance:**

This cohort study among First Nations people found low rates of OAT retention. Although retention was higher for methadone, it declined over time. These findings highlights important gaps in OAT provision for First Nations people that may be improved by investments into First Nations–led treatment programs that integrate traditional, land-based programs to better support people with opioid use disorder across Ontario.

## Key Points

**Question:**

What is the duration of treatment retention among First Nations people in Ontario, Canada, initiating methadone and buprenorphine-naloxone treatment and what factors are associated with retention?

**Findings:**

In this cohort study including 17 880 new courses of opioid agonist therapy (OAT), median duration of buprenorphine-naloxone (42 days) was shorter than methadone (71 days), with high health care utilization, differing rural/urban or First Nation–community residence, and comorbidity burden associated with OAT discontinuation. Methadone retention declined over calendar time.

**Meaning:**

These findings suggest that changes are needed to adapt OAT programs to meet the preferences and needs of First Nations people living in both rural and urban locations and those with high health care needs.

## Teaser

This cohort study examines factors associated with opioid agonist therapy retention within the first year of treatment among First Nations people in Ontario, Canada.

## Introduction

First Nations people are disproportionately impacted by the opioid crisis in Canada, experiencing higher rates of opioid use disorders (OUDs) and opioid-related morbidity and mortality.^1,2^ The impact of colonization, intergenerational trauma, anti-Indigenous racism, erosion of First Nations cultures, and barriers to accessing health care services have contributed to the increased risk of opioid-related harm among First Nations people.^1,3,4^ Between 2019 and 2021, the annual rate of opioid-related deaths among First Nations people in Ontario increased from 4.1 to 11.4 deaths per 10 000 population.^5^ Additionally, the rate of hospital visits for opioid-related toxic events more than doubled among First Nations people living within First Nation communities (2.6 to 6.2 events per 10 000 population) and tripled among First Nations people living outside of First Nation communities (8.4 to 26.8 events per 10 000 population) between the first quarter of 2018 and last quarter of 2021.^5^ To help address these increasing opioid-related harms, First Nations people and communities have made concerted efforts at broadening access to opioid agonist therapy (OAT) and harm reduction.

OAT is an evidence-based treatment for OUD associated with a greater than 50% reduction in the risk of all-cause mortality among people with OUD.^6,7^ In Ontario, methadone and buprenorphine-naloxone treatments are both considered first-line OAT and can be prescribed in both specialized OAT clinics and by primary care practitioners.^8,9^ However, despite the costs of these services being fully covered, historically entrenched structural barriers to care limit access to OAT among First Nations people in Canada. Most notably, a lack of OAT prescribers and limited pharmacy availability pose considerable barriers to treatment access among First Nations people living in remote and rural communities and those living within First Nation communities, resulting in lengthy travel and transportation challenges.^10^ In response, there have been ongoing efforts to expand access to OAT within First Nation communities, particularly those where access to pharmacies is limited. One such measure is the preferential use of buprenorphine-naloxone as OAT. Because it is considered safer than methadone, buprenorphine-naloxone permits more flexibility in take-home doses and requires fewer pharmacy visits.^5^ Accordingly, buprenorphine-naloxone is now the most commonly prescribed OAT among First Nations people in Ontario, despite methadone being the most commonly prescribed OAT in Ontario.^5^ However, studies suggest that retention to buprenorphine products can be low, with population-based studies in Ontario suggesting that buprenorphine-naloxone retention is inferior to methadone.^11,12^ However, to our knowledge, there are no complementary data examining rates of and factors associated with treatment retention to OAT among First Nations people in Ontario. As a result, this study investigates duration of treatment retention among First Nations people in Ontario, Canada, initiating methadone and buprenorphine-naloxone and describes the factors associated with treatment retention over an 8-year period that includes the COVID-19 pandemic.

## Methods

This cohort study was approved by the Unity Health Toronto Research Ethics Board with a waiver of informed consent because we used deidentified ICES data. The study was led by the Chiefs of Ontario (COO), in partnership with the Ontario Drug Policy Research Network. This work has been mandated by COO leadership through Resolution 20-18 Prescription Opioid Surveillance. Research staff members from COO and members of the Opioid Surveillance Steering Committee, representing First Nation communities across Ontario, provided advice and knowledge on the analysis plan, data interpretation and manuscript preparation. The research adheres to the First Nations principles of OCAP (ownership, control, access, and possession).^13^ On the advice of the Steering Committee, this analysis uses a strength-based approach by focusing on retention rates among First Nations people in Ontario and does not make comparisons to individuals who do not have registered First Nations status.^14,15,16,17^ This study follows the Consolidated Criteria for Strengthening Reporting of Health Research Involving Indigenous Peoples (CONSIDER) statement^18^ and Strengthening the Reporting of Observational Studies in Epidemiology (STROBE) reporting guideline.

### Setting and Data Sources

We conducted a population-based retrospective cohort study including all registered First Nations people aged at least 15 years newly prescribed buprenorphine-naloxone or methadone in Ontario between January 1, 2013, and March 31, 2021, with a follow-up period up to March 31, 2022. We used administrative health data at ICES, an independent, nonprofit research institute whose legal status under Ontario’s health information privacy allows it to collect and analyze health care and demographic data, without individuals’ consent, for health system evaluation and improvement. Use and sharing of the data are in accordance with a Data Governance Agreement between COO, in partnership with ICES, the First Nations and Inuit Health Branch, and Cancer Care Ontario, to access and transfer the Indian Registry System (IRS) data to ICES.^19^ For this study we used the IRS database to identify all registered (status) First Nations people in Ontario, which includes all people who are eligible and have registered for “Indian Status” under the Indian Act. We acknowledge and believe that language used in the legislation to describe First Nations people, such as *Indian*, is outdated, offensive, and rooted in colonialism.^20^

We used the Registered Persons Database (RPDB) to ascertain the demographic and geographic characteristics of all individuals and the Narcotics Monitoring System (NMS), which captures all prescriptions for controlled substances dispensed from community pharmacies in Ontario, to identify prescribing of opioids, benzodiazepines, and stimulants. We used the Canadian Institute for Health Information (CIHI) National Ambulatory Care Reporting System, CIHI Discharge Abstract Database, Ontario Mental Health Reporting System (OMHRS) and CIHI Same Day Surgery Database (SDS) for information on emergency department (ED) visits, inpatient hospitalizations, mental health hospitalizations and outpatient surgeries, respectively. The Ontario Health Insurance Plan claims database captured all outpatient physician visits, while validated ICES-derived cohorts identified diagnoses of HIV and diabetes.^21,22^ We used the ICES Physician Database to identify prescriber characteristics, including location of practice. All the datasets were linked using unique encoded identifiers and analyzed at ICES in Toronto, Ontario. To minimize the risk of reidentification, small counts (n < 6) were not reported.

### Cohort Definition and Exposure

We constructed separate cohorts of First Nations people initiating methadone and buprenorphine-naloxone, with the date of the first prescription serving as the index date. We defined new use as no dispenses for the cohort-specific treatment in the prior 30 days. We included all treatment courses for individuals initiating multiple courses during the study period. We excluded individuals with both OATs dispensed at the index date, individuals aged younger than 15 years or older than 105 years, non–Ontario residents, and individuals with missing sex or age or invalid patient identifiers. We did not include people with evidence of take-home doses at index, as this would imply ongoing treatment (eTable 1 in Supplement 1).

### Outcomes

Our primary outcome was duration of treatment retention for methadone and buprenorphine-naloxone. We defined treatment discontinuation as missing 14 consecutive days of OAT, a timeframe representing a clinically meaningful gap that requires a reinitiation protocol before continuing treatment.^11,23^ The treatment end date was the day on which the final dispensed OAT supply would run out. We censored follow-up on the first occurrence of switch to an alternative OAT, death, 365 days of follow-up, or end of data availability (March 31, 2022). For courses that ended in discontinuation, we identified dose tapering as a daily dose of 10 mg or less for methadone or 2 mg or less for buprenorphine-naloxone on the last prescription.

### Cohort Characteristics

We determined demographic characteristics for individuals receiving OAT treatment courses, including age, sex, community size and metropolitan influenced zones (eTable 2 in Supplement 1),^24^ living within or outside First Nation communities, residence in Northern Ontario, and neighborhood income quintile. We reported clinical characteristics potentially associated with OAT discontinuation, including the Charlson Comorbidity Index^25^ and diagnoses of HIV, diabetes, mental health conditions, and pain-related injuries (eTable 3 in Supplement 1). We recorded treating clinician characteristics for the initial OAT prescription, including the linear distance in kilometers to the prescriber and whether the prescription was written by a high volume OAT prescriber (top 5% of prescribing volume in that year). We also determined receipt of prior dispenses of OAT, non-OAT opioids, stimulants, and benzodiazepines in the 6 months preceding the index date (eTable 4 in Supplement 1). Finally, we reported prior health care system utilization, including ED visits (any and opioid-related toxic event visits), inpatient hospitalizations, and virtual and nonvirtual non-OUD physician visits in the previous 12 months (eTable 3 in Supplement 1).

### Statistical Analysis

We summarized the characteristics of First Nations people initiating methadone and buprenorphine-naloxone using descriptive statistics. We used absolute standardized differences to identify differences between groups, with values greater than 0.10 suggesting a meaningful difference.^26^ We constructed Kaplan-Meier survival curves to estimate duration of treatment retention for each OAT type. We examined factors associated with OAT discontinuation within 1 year of treatment initiation by fitting multivariable Cox proportional hazards models with robust standard errors to account for individuals who contributed multiple treatment courses (reported as adjusted hazard ratios [aHRs] with 95% CIs), censoring on all aforementioned factors described. All cohort variables were included in the model, with the exception of distance to OAT prescriber due to concerns regarding collinearity with Northern Ontario location of residence. All analyses were performed at ICES using SAS statistical software version 9.4 (SAS Institute) and used a 2-sided type 1 error rate of 5% to define statistical significance. Data were analyzed between October 2022 and June 2024.

## Results

### Cohort Characteristics

**Figure 1.**
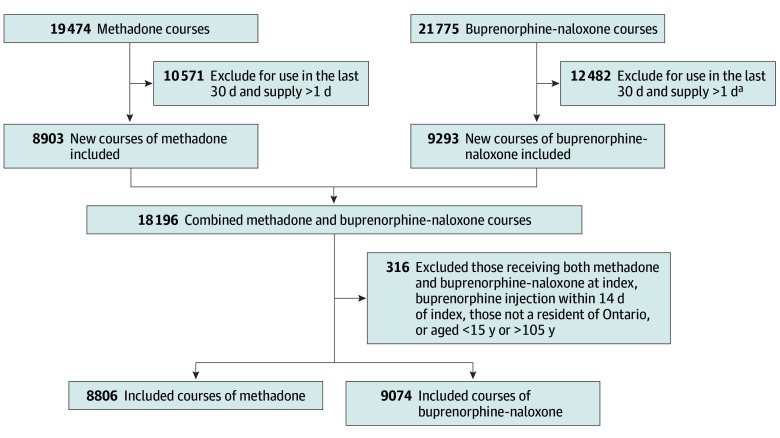
Flowchart of Cohort Inclusion and Exclusion Criteria Diagram showing the derivation of our final study cohort of 17 880 new courses of OAT based on inclusion and exclusion criteria. ^a^For buprenorphine-naloxone, initial days supply of 2 or 3 days included as long as all remaining days supply in first 14 days of treatment were of 1-day duration (to account for small numbers of take-home doses provided at initiation on holidays or weekends).

We studied 17 880 new courses of OAT among 7476 individuals (median [IQR] age, 31 [26-38] years; 8966 [50.1%] female) (Figure 1). Overall, 9704 new courses (50.8%) were for buprenorphine-naloxone (among 4869 individuals), and 8806 new courses (49.2%) were for methadone (among 4148 individuals) (Table 1). A higher percentage of individuals initiating methadone treatment lived outside First Nation communities compared with those initiating buprenorphine-naloxone (6848 individuals [77.8%] vs 5383 individuals [59.3%]; standardized difference, 0.40). Individuals starting buprenorphine-naloxone also lived further from their OAT prescriber compared with those initiating methadone (median [IQR] distance, 240 [23-639] km vs 42 [7-243] km; standardized difference, 0.45), and almost three-quarters of individuals initiating buprenorphine-naloxone lived in Northern Ontario (6614 individuals [72.9%]) compared with 3724 individuals (42.3%) starting methadone (standardized difference, 0.65) (Table 1).

**Table 1.** Characteristics of First Nations People Initiating OAT

Characteristic	Total OAT courses (n = 17 880), No. (%)		
	Buprenorphine-naloxone (n = 9074)	Methadone (n = 8806)	Standardized difference
Age, y			
Median (IQR),	31 (26-38)	31 (26-38)	0.02
15-24	1614 (17.8)	1416 (16.1)	0.05
25-34	4096 (45.1)	4278 (48.6)	0.07
35-44	2264 (25.0)	2129 (24.2)	0.02
45-64	1074 (11.8)	961 (10.9)	0.03
≥65	26 (0.3)	22 (0.2)	0.01
Sex			
Female	4577 (50.4)	4389 (49.8)	0.01
Male	4497 (49.6)	4417 (50.2)	0.01
Community size and MIZ^a^			
Very large urban (≥1 500 000)	395 (4.4)	774 (8.8)	0.18
Large urban area (500 000-1 499 999)	206 (2.3)	570 (6.5)	0.21
Medium urban area (100 000-499 999)	2417 (26.6)	3502 (39.8)	0.28
Small urban area (10 000-99 999)	889 (9.8)	1137 (12.9)	0.10
Rural with strong urban influence (strong MIZ)	208 (2.3)	394 (4.5)	0.12
Rural with moderate urban influence (moderate MIZ)	722 (8.0)	742 (8.4)	0.02
Rural with weak or no urban influence (weak MIZ)	4169 (45.9)	1583 (18.0)	0.63
Missing	68 (0.7)	104 (1.2)	0.04
Residence			
Within First Nations community	3686 (40.6)	1949 (22.1)	0.41
Outside First Nations community	5385 (59.3)	6848 (77.8)	0.40
Missing or unknown	<5 (0.0)	9 (0.1)	0.03
Residence in Northern Ontario	6614 (72.9)	3724 (42.3)	0.65
Distance from prescriber, median (IQR), km	240 (23-639)	42 (7-243)	0.45
Neighborhood income quintile			
1 (Lowest)	5863 (64.6)	4660 (52.9)	0.24
2	1054 (11.6)	1427 (16.2)	0.13
3	836 (9.2)	977 (11.1)	0.06
4	623 (6.9)	728 (8.3)	0.05
5 (Highest)	577 (6.4)	706 (8.0)	0.06
Missing	121 (1.3)	308 (3.5)	0.14
Charlson Comorbidity Index (previous 3 y)			
No hospitalizations	5272 (58.1)	5148 (58.5)	0.01
0	3020 (33.3)	2979 (33.8)	0.01
1	457 (5.0)	470 (5.3)	0.01
2	157 (1.7)	95 (1.1)	0.06
≥3	168 (1.9)	114 (1.3)	0.04
HIV diagnosis	103 (1.1)	171 (1.9)	0.07
Diabetes diagnosis	876 (9.7)	535 (6.1)	0.13
Mental health diagnoses (previous 3 y)	3640 (40.1)	3560 (40.4)	0.01
Schizophrenia	254 (2.8)	300 (3.4)	0.04
Mood disorders	500 (5.5)	453 (5.1)	0.02
Anxiety disorders	1116 (12.3)	1089 (12.4)	0
Deliberate self-harm	721 (7.9)	834 (9.5)	0.05
Other mental health diagnoses	195 (2.1)	197 (2.2)	0.01
OAT prescription (previous 6 mo)			
Methadone	1636 (18.0)	4072 (46.2)	0.63
Buprenorphine-naloxone	3618 (39.9)	1273 (14.5)	0.60
Buprenorphine extended release injection (subcutaneous)	14 (0.2)	10 (0.1)	0.01
Buprenorphine subdermal implant	0	0	NA
SROM (OAT use)	44 (0.5)	21 (0.2)	0.04
Prescribed medications (previous 6 mo)			
Opioids (non-OAT)	1232 (13.6)	1547 (17.6)	0.11
Stimulants	325 (3.6)	433 (4.9)	0.07
Benzodiazepines	808 (8.9)	961 (10.9)	0.07
Health system utilization (last 12 mo)			
High-volume index prescriber (top 5%)	5619 (61.9)	7062 (80.2)	0.41
ED visit for opioid-related toxic events	344 (3.8)	554 (6.3)	0.11
Nonvirtual physician visits (non-OUD), median (IQR), No.	8 (2-25)	11 (2-32)	0.14
Virtual physician visits (non-OUD), median (IQR), No.	0 (0-3)	0 (0-3)	0.01
≥1 ED visit	5462 (60.2)	5633 (64.0)	0.08
≥1 Inpatient hospitalization	1673 (18.4)	1534 (17.4)	0.03
Pain-Related Injuries and Conditions (previous 5 y)	6252 (68.9)	6513 (74.0)	0.11
Low back pain	3348 (36.9)	3589 (40.8)	0.08
Fractures, dislocations, strains, or sprains	4362 (48.1)	4600 (52.2)	0.08
Arthritis and related conditions	2567 (28.3)	2863 (32.5)	0.09
Bone and spinal conditions	2572 (28.3)	2701 (30.7)	0.05
Unspecified musculoskeletal disorders or congenital abnormalities	2929 (32.3)	3230 (36.7)	0.09
Traumatic brain injury^b^	728 (8.0)	792 (9.0)	0.03

Abbreviations: ED, emergency department; MIZ, Metropolitan Influence Zone; NA, not applicable; OAT, opioid agonist therapy; OUD, opioid use disorder; SROM, slow release oral morphine.

^a^ Further details on community size and MIZ are provided in eTable 2 in Supplement 1.

^b^ Reported in the previous 10 years.

### Duration of OAT Retention

**Figure 2.**
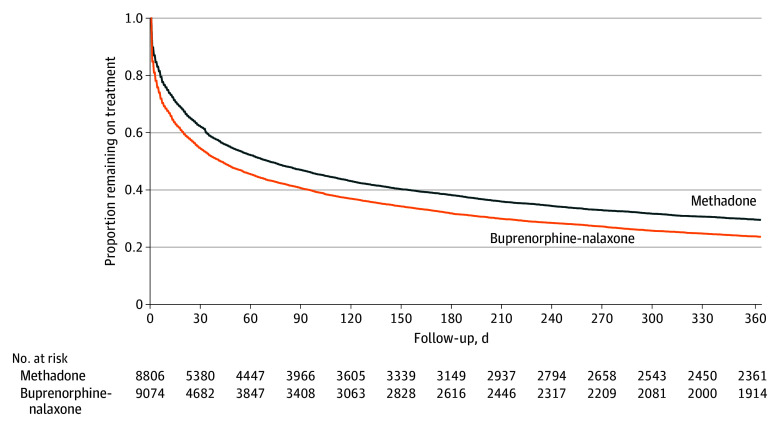
Kaplan-Meier Survival Curve for Opioid-Agonist Therapy Retention Kaplan-Meier survival curves are presented for new courses of methadone and buprenorphine-naloxone among First Nations people in Ontario during the first 365 days of treatment.

Among new methadone treatment episodes, the median (IQR) duration of treatment was 71 (10 to 544) days, with 38.0% of episodes ending within 30 days and 70.5% of episodes ending within 1 year (Figure 2). Among new buprenorphine-naloxone treatment episodes, the median (IQR) duration of treatment was significantly lower, at 42 (5 to 321) days (*P* < .001), with 45.7% of episodes ending within 30 days and 76.4% of episodes ending within 1 year. Only 641 new methadone treatment episodes (7.3%) and 630 new buprenorphine-naloxone treatment episodes (6.9%) were censored due to individuals switching to a different OAT during the follow-up period. Similarly, tapering of dose prior to discontinuation was uncommon, with 670 buprenorphine-naloxone discontinuations (8.8%) and 337 of 6605 methadone discontinuations with available data (5.1%) having evidence of tapered dosing (eTable 5 in Supplement 1).

### Factors Associated With OAT Retention

In multivariable analysis, several factors were associated with methadone treatment retention (Table 2). Specifically, First Nations individuals living outside of a First Nation community were more likely to discontinue OAT (aHR, 1.13; 95% CI, 1.04-1.23) and treatment discontinuation increased over calendar time (2021 vs 2013: aHR, 1.74; 95% CI, 1.47-2.06). Similarly, individuals residing in lower-income neighborhoods (income quintile 1 vs 5: aHR, 1.16; 95% CI, 1.04-1.31), those with an HIV diagnosis (aHR, 1.45; 95% CI, 1.14-1.83), and those diagnosed with schizophrenia at time of initiation (aHR, 1.23; 95% CI, 1.07-1.41) were more likely to discontinue methadone within 1 year, as were those who had recently been dispensed methadone (aHR, 1.67; 95% CI, 1.57-1.78), who had recently visited an ED for any cause (aHR, 1.22; 95% CI, 1.15-1.30) or for an opioid toxic event (aHR, 1.24; 95% CI, 1.11-1.38), and those recently hospitalized (aHR, 1.16; 95% CI, 1.06-1.26). Factors associated with improved methadone retention included being aged 45 to 64 years (aHR, 0.82; 95% CI, 0.72-0.93), residing in moderately sized urban areas (population 100 000 to 499 999 vs ≥1 500 000: aHR, 0.79; 95% CI, 0.70-0.90), being recently prescribed buprenorphine-naloxone (aHR, 0.88; 95% CI, 0.82-0.95) or non-OAT opioids (aHR, 0.86; 95% CI, 0.79-0.93), and having higher numbers of non-OUD-related physician visits in the year prior.

**Table 2.** Factors Associated With Treatment Discontinuation Among First Nations People Receiving OAT Stratified by Therapy Type

Factor	Adjusted hazard ratio (95% CI)	
	Buprenorphine-naloxone	Methadone
Residence		
Living inside First Nation community	1 [Reference]	1 [Reference]
Living outside First Nation community	0.89 (0.82-0.97)^a^	1.13 (1.04-1.23)^a^
Year		
2013	1 [Reference]	1 [Reference]
2014	1.01 (0.85-1.19)	1.17 (1.03-1.32)^a^
2015	1.04 (0.89-1.22)	1.18 (1.04-1.34)^a^
2016	0.93 (0.80-1.09)	1.26 (1.11-1.42)^a^
2017	1.01 (0.86-1.17)	1.30 (1.15-1.48)^a^
2018	0.97 (0.83-1.13)	1.48 (1.30-1.68)^a^
2019	1.01 (0.86-1.17)	1.71 (1.51-1.94)^a^
2020	0.83 (0.71-0.97)^a^	1.75 (1.54-1.98)^a^
2021	1.01 (0.84-1.22)	1.74 (1.47-2.06)^a^
Age category, y		
15-24 y	1 [Reference]	1 [Reference]
25-34	1.00 (0.92-1.08)	1.08 (1.00-1.17)
35-44	0.97 (0.88-1.07)	0.99 (0.90-1.09)
45-64	0.85 (0.75-0.97)^a^	0.82 (0.72-0.93)^a^
≥65	0.95 (0.69-1.31)	1.84 (0.70-4.84)
Sex	1.00	1.00
Women	1 [Reference]	1 [Reference]
Men	0.98 (0.92-1.05)	0.94 (0.88-1.01)
Community size and metropolitan influence zones		
500 000-1 499 999	0.89 (0.72-1.10)	0.94 (0.80-1.10)
100 000-499 999	0.81 (0.70-0.95)^a^	0.79 (0.70-0.90)^a^
10 000-99 999	0.97 (0.83-1.15)	0.87 (0.75-1.00)
≥1 500 000	1 [Reference]	1 [Reference]
Rural with strong urban influence	0.74 (0.59-0.93)^a^	0.75 (0.60-0.95)^a^
Rural with moderate urban influence	0.84 (0.71-1.00)	0.85 (0.73-1.00)
Rural with weak or no urban influence	0.71 (0.60-0.84)^a^	0.91 (0.78-1.07)
Northern residence	0.93 (0.86-1.01)	1.00 (0.93-1.08)
Income quintile		
1 (Lowest)	1.03 (0.92-1.16)	1.16 (1.04-1.31)^a^
2	0.98 (0.86-1.12)	1.15 (1.01-1.31)^a^
3	1.01 (0.87-1.16)	1.01 (0.87-1.17)
4	1.03 (0.88-1.21)	0.99 (0.85-1.16)
5 (Highest)	1 [Reference]	1 [Reference]
Charlson category		
No hospitalizations	1 [Reference]	1 [Reference]
0	1.02 (0.94-1.09)	1.03 (0.96-1.11)
1	1.32 (1.11-1.57)^a^	1.17 (1.02-1.35)^a^
2	1.53 (1.19-1.96)^a^	1.37 (0.96-1.96)
≥3	1.51 (1.20-1.92)^a^	1.18 (0.87-1.60)
HIV diagnosis	1.38 (1.03-1.84)^a^	1.45 (1.14-1.83)^a^
Diabetes diagnosis	1.02 (0.88-1.19)	0.92 (0.79-1.07)
Mental Health diagnoses		
Schizophrenia	1.16 (1.00-1.35)	1.23 (1.07-1.41)^a^
Mood	1.01 (0.89-1.14)	1.08 (0.95-1.22)
Anxiety	1.01 (0.93-1.10)	1.02 (0.93-1.12)
Deliberate self-harm	1.08 (0.97-1.19)	1.07 (0.98-1.18)
Other	1.16 (0.97-1.38)	0.93 (0.77-1.13)
Medications in previous 6 mo		
Methadone	1.09 (1.01-1.18)^a^	1.67 (1.57-1.78)^a^
Oral Buprenorphine/Naloxone	1.32 (1.24-1.40)^a^	0.88 (0.82-0.95)^a^
Buprenorphine Extended Release injection (s/c)	1.13 (0.72-1.78)	0.73 (0.27-1.97)
SROM (OAT use)	0.62 (0.41-0.92)^a^	1.18 (0.81-1.73)
Non-OAT opioids	0.86 (0.80-0.94)^a^	0.86 (0.79-0.93)^a^
Stimulants	0.98 (0.86-1.12)	0.96 (0.85-1.07)
Benzodiazepines	0.92 (0.84-1.02)	0.98 (0.89-1.09)
Health care utilization		
High-volume prescriber	0.84 (0.79-0.89)^a^	1.00 (0.92-1.08)
ED visit for opioid-related toxic event	1.36 (1.20-1.54)^a^	1.24 (1.11-1.38)^a^
Number of nonvirtual non-OUD physician visits^b^	0.96 (0.95-0.96)^a^	0.96 (0.96-0.97)^a^
Number of virtual non-OUD physician visits^b^	0.99 (0.97-1.01)	0.97 (0.95-0.98)^a^
ED visit for any reason	1.10 (1.03-1.18)^a^	1.22 (1.15-1.30)^a^
Hospitalization for any reason	1.08 (0.99-1.18)	1.16 (1.06-1.26)^a^
Pain-related injuries and other conditions		
Traumatic brain injury	1.13 (1.01-1.26)^a^	1.01 (0.91-1.12)
Low back pain	1.04 (0.95-1.13)	0.92 (0.85-1.00)
Fracture, dislocations, strains, or sprains	1.00 (0.93-1.07)	1.01 (0.94-1.08)
Arthritis and related conditions	1.02 (0.95-1.09)	1.02 (0.95-1.09)
Bone and spinal conditions	0.96 (0.88-1.05)	0.98 (0.89-1.07)
Unspecified musculoskeletal disease	1.09 (1.02-1.17)^a^	1.05 (0.98-1.12)

Abbreviations: ED, emergency department; OAT, opioid agonist therapy; OUD, opioid use disorder; SROM, slow release oral morphine.

^a^ *P* < .05.

^b^ Grouped by 5 (eg, 0 = 0; 1-5 = 1; 6-10 = 2).

While many factors were similarly associated with buprenorphine-naloxone retention, several differences were identified (Table 2). Specifically, in contrast to methadone, residing outside of a First Nation community was associated with a lower risk of buprenorphine-naloxone discontinuation (aHR, 0.89; 95% CI, 0.82-0.97), as was residing in a rural region with weak or no urban influence (aHR, 0.71; 95% CI, 0.60-0.84). Treatment discontinuation was not associated with income quintile, schizophrenia diagnosis, or calendar time, with the exception of 2020, when a significantly lower discontinuation was identified compared with 2013 (aHR, 0.83; 95% CI, 0.71-0.97). In contrast to what was observed among new methadone episodes, prior methadone and buprenorphine-naloxone treatment was associated with a higher risk of buprenorphine-naloxone discontinuation (prior methadone use: aHR, 1.09; 95% CI, 1.01-1.18; prior buprenorphine-naloxone use: aHR, 1.32; 95% CI, 1.24-1.40), and prior slow-release oral morphine use was associated with a lower risk of discontinuation (aHR, 0.62; 95% CI, 0.41-0.92). Finally, being initiated on buprenorphine-naloxone by a high-volume prescriber was associated with improved OAT retention (aHR, 0.84; 95% CI, 0.79-0.89), an association which was not observed among methadone treatment episodes.

## Discussion

This population-based study of 17 880 new OAT treatment episodes among 7476 First Nations people in Ontario, Canada, found that treatment retention was low with both methadone and buprenorphine-naloxone. Importantly, although OAT retention was higher among those initiating methadone compared with buprenorphine-naloxone (median time to discontinuation, 71 days vs 42 days), retention on methadone declined over time, a finding that may reflect inadequate methadone dosing among people exposed to the increasingly potent unregulated drug supply.^27^ Furthermore, dose tapering at time of discontinuation was uncommon, suggesting that individuals did not have a planned treatment discontinuation. Estimates of OAT retention in the overall Ontario population using similar methods are generally higher, with median treatment durations of 104 days and 265 days for buprenorphine-naloxone and methadone, respectively.^11^ Therefore, our findings demonstrate that First Nations people initiating OAT face ongoing and unique challenges to treatment retention, which likely include structural barriers to care and persistent health service access inequities that are legacies of colonization.

Our study identified several factors associated with treatment retention, including location of residence, comorbidity status, and prescriber experience with OAT. For example, First Nations people living in the largest urban centers were more likely to discontinue OAT, a finding that may reflect disparities in access to culturally appropriate, First Nations–led treatment options and community-based programs in large cities. Interestingly, we also found that retention was higher among buprenorphine-naloxone recipients residing in highly rural and remote parts of the province. This finding may reflect the improved safety profile of buprenorphine-naloxone relative to methadone, allowing for a more rapid transition to take-home dosing, circumventing barriers associated with observed doses, such as limited access to pharmacies in remote communities. For these reasons, expanding buprenorphine-naloxone availability to First Nations people in rural and remote regions of Ontario has been a priority for communities, policy makers, and clinicians.^10,28,29^ Our findings suggest that these efforts have likely been successful, with nearly one-half of all new buprenorphine-naloxone recipients residing in highly rural regions. Moreover, these individuals were retained on treatment longer than their counterparts residing in large urban centers. However, with more than half of First Nations people discontinuing treatment in the first 3 months of therapy, particularly among those residing within First Nation communities and large urban centers, additional efforts to improve treatment accessibility, acceptability, and retention among First Nations individuals are needed. These may include expanding the role of peer support and land-based programming in treatment provision; resourcing alternative treatment provision models, such as mobile outreach and medication dispensing; and integrating wraparound services that address other concurrent health diagnoses and social determinants of health. In particular, these services must be responsive to the ongoing impacts of structural violence, poverty, and trauma for First Nations people, ensuring adequate access to trauma-informed, culturally safe, First Nations–led substance use services throughout the province.^30,31,32^ Studies have highlighted the benefits of First Nations community–based treatment programs, with Ontario programs combining traditional healing with buprenorphine-naloxone reporting higher retention rates when compared with most OAT programs.^28^

Our findings also suggest that individuals living with HIV, multiple comorbidities, and those with a history of prior opioid toxic events were more likely to discontinue OAT, a finding that aligns with past studies of OAT retention.^12^ These patterns are likely driven by challenges faced by people with multiple concurrent diagnoses when managing frequent health care visits for other conditions. These barriers are generally greater for those residing in more rural regions and those with poor access to primary care. In our study, more than 40% of First Nations people starting OAT resided in rural parts of Ontario, and evidence suggests gaps exist in access to regular health care practitioners for First Nations people across Canada.^33^ Therefore, efforts to improve access to primary care practitioners for First Nations people and integrating treatment for OUD within these settings could enhance OAT retention and improve the general health of First Nations people across the province.

### Limitations

Our study has some limitations. First, we did not have data on nonpharmacologic treatments for substance use disorder provided throughout the province. Therefore, we are unable to determine the impacts of access to these services on OAT retention. We were also unable to include some other factors that may be associated with OAT retention, including access to First Nations–led health care services and/or harm reduction programs, Two Spirit identity, and roles supporting children and other dependents. Third, we focused our analysis on the 2 most commonly prescribed treatments for OUD, and did not include more recently introduced treatment options, such as buprenorphine injections or implants and slow-release oral morphine. However, because newer buprenorphine formulations were not approved by Health Canada until early 2020^34^ and guidance for using slow-release oral morphine for OAT was released in 2021,^7^ it is unlikely that this had a major impact on our findings. Fourth, we identified First Nations people using the IRS database, and therefore First Nations people who are not registered under Canada’s Indian Act are not included.

## Conclusions

In this cohort study, we found low rates of treatment retention to OAT among First Nations people initiating methadone or buprenorphine-naloxone, with evidence of improved retention in less populated regions. We also found more rapid discontinuation of methadone, particularly as fentanyl has dominated the unregulated drug supply. Our findings highlight the need for clinicians to adapt the provision of OAT to the preferences and needs of First Nations people living in urban, rural and remote locations, and to work closely with First Nations people to develop community-based, culturally appropriate strengths-based programs that support OAT initiation, retention, and when desired, tapering and discontinuation of treatment.

## Notes

Supplement 1. **eTable 1.** Exclusions of Take-Home Doses Used in This Study for Methadone and Buprenorphine-Naloxone Treatments **eTable 2.** Definition of Census Metropolitan Influenced Zone Variable Used to Categorize Location of Residence **eTable 3.** Codes Used to Define Patient Characteristics Using Administrative Data **eTable 4.** Definitions of High Volume OAT Prescriber and Any OAT in Previous 6 Months **eTable 5.** Tapered Discontinuation for Methadone and Buprenorphine-Naloxone

Supplement 2.
Data Sharing Statement
