# Supplementary material for: Retention in Opioid Agonist Therapy Among First Nations People
Source: JAMA Netw Open. 2025 Jun 30;8(6):e2518452. doi: 10.1001/jamanetworkopen.2025.18452 (PMC12210085; doi:10.1001/jamanetworkopen.2025.18452)
Supplement: Supplement 1. — eTable 1. Exclusions of Take-Home Doses Used in This Study for Methadone and Buprenorphine-Naloxone Treatments eTable 2. Definition of Census Metropolitan Influenced Zone Variable Used to Categorize Location of Residence eTable 3. Codes Used to Define Patient Characteristics Using Administrative Data eTable 4. Definitions of High Volume OAT Prescriber and Any OAT in Previous 6 Months eTable 5. Tapered Discontinuation for Methadone and Buprenorphine-Naloxone [file jamanetwopen-e2518452-s001.pdf]

Supplemental Online Content

Holton A, Hamzat B, McCormack D, et al. Retention in opioid agonist therapy among First Nations people. *JAMA Netw Open*. 2025;8(7):e2518452.
doi:10.1001/jamanetworkopen.2025.18452

eTable 1. Exclusions of Take-Home Doses Used in This Study for Methadone and Buprenorphine-Naloxone Treatments
eTable 2. Definition of Census Metropolitan Influenced Zone Variable Used to Categorize Location of Residence
eTable 3. Codes Used to Define Patient Characteristics Using Administrative Data
eTable 4. Definitions of High Volume OAT Prescriber and Any OAT in Previous 6 Months
eTable 5. Tapered Discontinuation for Methadone and Buprenorphine-Naloxone

This supplemental material has been provided by the authors to give readers additional information about their work.

© 2025 Holton A et al. *JAMA Network Open*.

eTable 1: Exclusions of take-home doses used in this study for methadone and buprenorphine/naloxone treatments

**Methadone**	We excluded individuals starting methadone with evidence of take home doses (i.e. multiple dispenses on index-date).
**Buprenorphine/naloxone**	Among new buprenorphine/naloxone recipients, we applied the same exclusion but did not exclude people who received 2 or 3 take-home doses at index with no evidence of additional take-home doses in the following 14 days to allow for potential home induction where first dose(s) are given as take-home doses. We applied this criteria because during the study period Canadian guidelines recommended witnessed dosing for buprenorphine/naloxone after home induction [35].<br><br>Additionally, we excluded courses where a buprenorphine extended-release (ER) injection was dispensed on the index date or 14 days thereafter, to exclude individuals receiving buprenorphine/naloxone during the induction for this product.

eTable 2: Definition of Census metropolitan influenced zone (CSizeMIZ) variable used to categorise location of residence[24]

Category	Definition
Very Large Urban	1,500,000+ population size
Large Urban Area	500,000-1,499,999 population size
Medium Urban Area	100,000-499,999 population size
Small Urban Area	10,000-99,999 population size
Rural with strong urban influence (strong MIZ)	Strong MIZ category includes census subdivisions (CSD) in provinces where ≥ 30% of the resident employed labour force works in any census metropolitan areas (CMAs) and census agglomeration (CA) core. It excludes CSDs with fewer than 40 persons in their resident employed labour force from the previous census.
Rural with moderate urban influence (moderate MIZ)	Moderate MIZ at least 5%, but <30% of the resident employed labour force works in any CMA or CA core. It excludes CSDs with fewer than 40 persons in their resident employed labour force from the previous census.
Rural with weak or no urban influence (weak MIZ) and no MIZ	Weak MIZ: > 0%, but <5% of the resident employed labour force works in any CMA or CA core and It excludes CSDs with fewer than 40 persons in their resident employed labour force from the previous census.<br><br>No MIZ category includes CSDs in provinces where none of the CSD's resident employed labour force (excluding the category of no fixed workplace address) commute to work in any delineation core of a CMA or CA. It also includes CSDs in provinces with fewer than 40 persons in their resident employed labour force from the previous census.

© 2025 Holton A et al. *JAMA Network Open*.

eTable 3: Codes used to define patient characteristics using administrative data

Condition	Data sources	Codes
Arthritis and related conditions (including inflammatory arthritis, other arthritis, soft tissue disorders, joint derangement, unspecified arthritis)* (previous 5 years)	OHIP, DAD, NACRS	NACRS and DAD databases:<br>*ICD-10*: M05-M09, M45, M46, M30-M36, M15-M19, M00-M03, M12, M10, M11, M14, M65-M71, M60-M63, M72-M73, M75-M77, M79, M22-M24, M13, M96, M99<br><br>OHIP:<br>*Dxcodes:* 714, 720, 710, 446, 711, 715, 716, 274, 712, 727, 728, 729, 717, 718, 739
Anxiety disorders (previous 3 years)	National Ambulatory Care Reporting System, Discharge Abstract Database, Ontario Mental Health Reporting System	ICD-10 diagnosis codes: F40, F41, F42, F43, F48.8, F48.9, F93.1, F93.2<br>DSM diagnosis codes: 300, 300.0, 300.2, 300.3, 308.3, 309.0, 309.24, 309.28, 309.3, 309.4, 309.8, 309.9.<br>Ontario Mental Health Reporting System Provisional Diagnosis: 7, 15
Charlson Comorbidity Index (previous 3 years)	DAD	
Deliberate self-harm (previous 3 years)	National Ambulatory Care Reporting System, Discharge Abstract Database	ICD-10 diagnosis codes: X60-X84, Y10-Y19, Y28 where main problem or most responsible diagnosis is not one of the ICD-10 diagnosis codes between F06 and F99
Diabetes	ICES-derived cohort	
Fractures, dislocations, strains or sprains *<br>(previous 5 years)	OHIP, DAD, SDS, NACRS	***ICD-10***: T08, S12, S32, S42, S52, S62, S72, S82, S92, S13.0-S13.3, S33.0-S33.3, S43.0-S43.3, S53.0-S53.1, S63.0-S63.2, S73.0, S83.0-S83.1, S93.0-S93.3, S13.4, S13.5, S33.5-S33.7 S43.4-S43.7, S53.2-S53.4, S63.3-S63.7, S73.1, S83.2-S83.7, S93.4-S93.6<br><br>**OHIP:** ***Dxcodes***: 802, 803, 805, 806, 807, 808, 810, 812, 813, 814, 815, 816, 821, 823, 824, 827, 829, 831, 832, 834, 839, 840, 841, 842, 843, 844, 845, 847, 848
Health service use (last 12 months)<br>• ED visit for opioid-related toxicity	DAD, NACRS, OHIP	Hospitalizations: Count unique episodes (EPI)<br><br>Physician visits:<br>Only count 1 outpatient physician visit per day<br>Do not include the following feecodes: K682 K683 K684 A957 K680 G040 G041 G042 G043.

© 2025 Holton A et al. *JAMA Network Open*.

• Number of non-virtual physician visits (non-OUD)<br>• Number of virtual physician visits (non-OUD)<br>• ED visits<br>• Hospitalizations		
Human immunodeficiency virus	ICES-derived cohort	
Low back pain * <br>(previous 5 years)	OHIP, DAD, SDS, NACRS	NACRS, SDS, and DAD: *ICD-10:* M4726, M4727, M4728, M4786, M4787, M4788, M4796, M4797, M4798, M4806, M4807, M4808, M4886, M4887, M4888, M4896, M4897, M4898, M510, M511, M512, M513, M519, M533, M5386, M5387, M5388, M5410, M5416, M5417, M5418, M5419, M543, M544, M545, M548, M549, M9983, M9993, M9903, M9984, M9994, M9904, S335, S336, S337, S338 (Wong et al., 2021)<br><br>DAD and SDS *CCI:* 3SC10KM, 3SC10VA, 3SC10VN, 3SE10VK, 3SC12AY, 3SC12VA, 3SE12VA, 3SE12VK, 3SF12VA, 3SF12VL, 3SF10VA, 3SF10VL (Wong et al., 2021)<br><br>OHIP: *Dxcode:* 722, 724, 847 (Health Quality Ontario, 2019; Wong et al., 2021)<br>*Feecode:* X025, X202, X203, X027, X204, X028, X205, X206, X032, X033, X031, X034, X207, X035, X208 (Wong et at., 2021)
Mood disorders (previous 3 years)	National Ambulatory Care Reporting System, Discharge Abstract Database, Ontario Mental Health Reporting System	ICD-10 diagnosis codes: F30, F31, F32, F33, F34, F38, F39, F53.0<br><br>DSM diagnosis codes: 296, 300.4, 301.13<br><br>Ontario Mental Health Reporting System Provisional Diagnosis: 6
Opioid-related Toxicity (last 12 months)	National Ambulatory Care Reporting System, Discharge Abstract Database	ICD-10 diagnosis codes: T400, T401, T402, T403, T404, T406
Other mental health disorders (previous 3 years)	National Ambulatory Care Reporting System, Discharge Abstract Database, Ontario	ICD-10 diagnosis codes: Any other diagnosis code between F06 and F99 not included in the other mental health-related categories, excluding dementia and delirium-related diagnoses.<br><br>DSM diagnosis codes: Any DSM code not included in

© 2025 Holton A et al. *JAMA Network Open*.

	Mental Health Reporting System	the other mental health-related categories, excluding dementia and delirium-related diagnoses.
Physician visits for opioid use disorder (last 12 months)	OHIP	OHIP Feecode: K682 K683 K684 A957 K680 G040 G041 G042 G043
Schizophrenia spectrum and other psychotic disorders (previous 3 years)	National Ambulatory Care Reporting System, Discharge Abstract Database, Ontario Mental Health Reporting System	ICD-10 diagnosis codes: F20 (excluding F20.4), F22, F23, F24, F25, F28, F29, F53.1<br>DSM diagnosis codes: 295, 297, 298<br>Ontario Mental Health Reporting System Provisional Diagnosis: 5
Tramuatic brain injury (TBI)* (previous 10 years)	DAD, NACRS	*ICD-10*: S02.0, S02.1, S02.3, S02.7, S02.8, S02.9, S06, S07.1, T90.2, T90.5

***Defined as pain related conditions**

eTable 4: Definitions of High Volume OAT prescriber and any OAT in previous 6 months:

Term	Definition
**High volume OAT Prescriber**	Those in the top 5% of prescribing volume based on a dataset of physician and nurse prescribers that includes the number of OAT patients they prescribed for in that year.
**Any OAT prescription in the previous 6 months**	Includes: Methadone, buprenorphine/naloxone, buprenorphine ER injection, buprenorphine implant and slow release oral morphine (SROM) in the previous 6 months

© 2025 Holton A et al. *JAMA Network Open*.

eTable 5: Tapered Discontinuation for Methadone and Buprenorphine/Naloxone

Treatment Type	Tapered Discontinuation
**Methadone**	Among methadone treatment courses with available dosage data (N=6,605), approximately one in twenty (5.1%; N=337) had tapered discontinuation i.e. received a daily dose of ≤10 mg.
**Buprenorphine/naloxone**	Just under one in ten buprenorphine/naloxone treatment courses (8.8%; N=670) had tapered discontinuation i.e. ≤2mg.

© 2025 Holton A et al. *JAMA Network Open*.
