# Supplementary material for: Retention in Opioid Agonist Therapy Among First Nations People
Source: JAMA Netw Open. 2025 Jun 30;8(6):e2518452. doi: 10.1001/jamanetworkopen.2025.18452 (PMC12210085; doi:10.1001/jamanetworkopen.2025.18452)
Supplement: Supplement 2. — Data Sharing Statement [file jamanetwopen-e2518452-s002.pdf]

Data Sharing Statement

Holton. Retention in Opioid Agonist Therapy Among First Nations People. *JAMA Netw Open*. Published July 01, 2025. doi:10.1001/jamanetworkopen.2025.18452

Data
Data available: No

Additional Information
Explanation for why data not available: The dataset from this study is held securely in coded form at ICES. While legal data sharing agreements between ICES and data providers (e.g., healthcare organizations and government) prohibit ICES from making the dataset publicly available, access may be granted to those who meet pre-specified criteria for confidential access, available at www.ices.on.ca/DAS (email: das@ices.on.ca). The full dataset creation plan is available from the authors upon request.
